# Supplementary material for: Non-Diabetic Kidney Disease in Type 2 Diabetes Mellitus: A Changing Spectrum with Therapeutic Ascendancy
Source: J Clin Med. 2023 Feb 20;12(4):1705. doi: 10.3390/jcm12041705 (PMC9964578; doi:10.3390/jcm12041705)
Supplement: Supplementary file 1 [file jcm-12-01705-s001.zip › jcm-2196615-supplementary.pdf]

Supplemental material table of contents

I. Section S1. Standard working definitions and classifications

II. Supplementary Table S1. Comparison of histopathological features

III. Supplementary Table S2. Histopathological findings of DKD on renal biopsy – DKD versus NDKD plus DKD

I. Section S1. **Standard working definitions and classifications**

I (A). Diagnosis of Diabetes Mellitus:

Defined as per the ADA' 2011 criteria i.e., presence of HbA1C ≥6.5% or Fasting blood glucose ≥126 mg/dL or 2-hour post prandial blood glucose ≥200 mg/dL or random blood glucose ≥200 mg/dL in a patient with classic symptoms of hyperglycemia or hyperglycemic crisis (10).

I (B). Definition of Type-1 Diabetes Mellitus (11 -13):

Patients with Diabetes Mellitus are considered to be Type-1 if they have the following features

1. Onset at young age < 20 years
2. Onset at the time of diagnosis of Diabetes Mellitus: Acute and severe at onset and/ or presence of diabetic ketoacidosis (DKA) at the time of onset.
3. History of episodes of DKA during the course
4. Body habitus: Lean/ thin built
5. Insulin dependency
6. Low fasting insulin/ C-peptide levels or presence of anti-GAD65 antibodies or anti-insulin antibodies (done in patients with inconclusive type based on clinical features).

I (C). Acute kidney injury (AKI):

AKI is defined as any of the following (14)

1. Increase in serum creatinine by ≥0.3 mg/dl within 48 hours (or)
2. Increase in serum creatinine to ≥1.5 times baseline, which is known or presumed to have occurred within the prior 7 days (or)
3. Urine volume <0.5 ml/kg/h for 6 hours.

I (D). Rapid decline in GFR:

Decline in GFR at a rate of > 5ml/min/1.73m^2 per year is defined as rapid decline in GFR as per the KDIGO definition (15)

I (E). Rapidly progressive renal failure: (16)

A syndrome of the kidney that is characterized by a rapid loss of kidney function (usually a 50% decline in the glomerular filtration rate (GFR) within 3 months)

I (F). Diabetic retinopathy (17)

Presence of any one of the following characteristic group of lesions found in the retina of individuals having had DM

1. Mild non-proliferative DR: Presence of only microaneurysms
2. Moderate non-proliferative DR: More than just microaneurysms but less than severe non-proliferative DR
3. Severe non-proliferative DR:

   Any of the following:

   - More than 20 intraretinal haemorrhages quadrants
   - Definite venous beading in 2+ quadrants - prominent intraretinal microvascular abnormalities in 1+ quadrant.
   - No signs of proliferative DR
4. Proliferative DR:

   One or more of the following:

   - Neovascularization
   - Vitreous/ pre-retinal haemorrhage
5. No DR: None of the above changes

I (G). Hypertension (18)

Hypertension was considered if the patients were receiving anti-hypertensives or if the patients had a systolic pressure of >130 mmHg and diastolic blood pressure> 80 mmHg

I (H). Coronary artery disease (19):

The patients were considered to be suffering from coronary artery disease, if they had a past or present history of acute coronary syndrome or, chronic stable angina requiring medical, endovascular, or surgical interventions, including antiplatelet therapy or thrombolysis, percutaneous stenting, and coronary artery bypass surgery.

I (I). Cerebrovascular accident (20):

Defined as brain, spinal cord, or retinal cell death attributable to ischemia, based on1. pathological, imaging, or other objective evidence of cerebral, spinal cord, or retinal focal ischemic injury in a defined vascular distribution; or2. clinical evidence of cerebral, spinal cord, or retinal focal ischemic injury based on symptoms persisting ≥24 hours or until death, and with other aetiologies excluded.

I (J). Diabetic neuropathy:

Presence of symptoms and/or signs of peripheral nerve dysfunction in patients with diabetes after the exclusion of other causes. (21)

I (K). Peripheral artery disease:

Arterial stenosis or occlusion causing an imbalance of blood flow relative to the metabolism either presenting as claudication/ rest pain in the limbs, ulcers over the pressure sores or acute critical limb ischemia. (22)

I (L). Diagnosis and classification of diabetic kidney disease:

The pathologic criteria for DKD included diffuse mesangial matrix expansion and glomerular basement membrane thickening (≥450 nm) with or without the presence of nodular mesangial deposits. Other supportive biopsy findings of DKD included thickening of the viable tubular basement membranes, lesions associated with hyaline deposits including arteriolar hyalinosis, fibrin caps and capsular drops, diffuse linear staining of IgG on glomerular and tubular basement membranes on immunofluorescence. A diagnosis of acute tubulointerstitial nephritis (ATIN) was made only if interstitial infiltrates involved non-fibrotic areas, included eosinophils, and displayed evidence of tubular inflammation. A diagnosis of acute tubular necrosis (ATN) was made if any of the following findings like simplification of tubular epithelium, loss of brush border, thyroidization were seen in viable, non-atrophic tubules (23 – 25).

I (M). Diagnosis of Non-diabetic kidney disease:

Histological diagnosis of various NDKD including IgA Nephropathy(IgAN), Focal-segmental glomerulosclerosis(FSGS), minimal change disease(MCD), Membranous nephropathy, cast nephropathy, various deposition diseases including amyloidosis, monoclonal immune deposition diseases(MIDD), Granulomatous interstitial nephritis(GIN), crescentic glomerulonephritis(pauci-immune or anti-GBM related) were made based on the standard criteria on light microscopy, immunofluorescence and electron microscopy (23).

i. Infection related glomerulonephritis, patients were diagnosed as infection related glomerulonephritis (IRGN) if they fulfilled 3 out of 5 criteria (26, 27).

- Presence of clinical or laboratory evidence of infection preceding or at the onset of glomerulonephritis
- Decreased serum C3 complement level
- Endocapillary proliferative and exudative glomerulonephritis

- C3-dominance or co-dominance on immunofluorescence staining in glomeruli
- Presence of subepithelial humps on electron microscopy

ii. The diagnosis of IgA Dominant IRGN was considered if following features are present (28,29):

- Initial presentation in older age
- Acute Kidney Injury presentation
- Intercurrent staphylococcal infection
- Presence of hypocomplementemia
- Prominent glomerular neutrophil infiltration on light microscopy
- Dominant of co-dominant glomerular IgA deposits on immunofluorescence, and
- Presence of subepithelial hump-shaped deposits on Electron microscopy

iii. If endocapillary proliferation is associated with chronic changes including GBM reduplication/ lobular accentuation, patients are diagnosed under the category of MPGN. Other supportive features considered while making a diagnosis of MPGN include (30):

- Lack of clinical evidence of infection
- Persistently low C3 for longer than several months
- Persistently active glomerulonephritis for more than several months
- Large mesangial, intramembranous, or subendothelial deposit

Further sub-classification of MPGN into immune-complex associated or complement associated was based on the deposits on immunofluorescence examination as per the recent classification (30).

II. Supplementary Table S1. Comparison of Vascular, tubule-interstitial changes and immunofluorescence pattern on histopathology between DKD and NDKD groups.

Parameter	DKD (n=166)	NDKD (n=372)	p-value
Degree of IFTA			
- **No (<10%)**	1 (0.6%)	59 (15.8%)	**<0.001**
- **Mild**	64 (38.6%)	168 (45.2%)	0.15
- **Moderate**	78 (47%)	111 (29.8%)	**< 0.001**
- **Severe**	23 (13.9%)	34 (9.1%)	0.1
Vascular changes			
- **No Hyalinosis**	12 (7.2%)	145 (38.9%)	**< 0.001**
- **Both afferent and efferent arteriolar Hyalinosis**	140 (84.3%)	89 (23.9%)	**< 0.001**
- **Only Afferent arteriolar hyalinosis**	14 (8.4%)	138 (37%)	**< 0.001**
Dominant Immunofluorescence pattern			
- **No deposits**	77 (46.4%)	43 (11.5%)	**< 0.001**
- **IgG deposits**	32 (19.2%)	59 (15.8%)	0.33
- **IgM**	26 (14.4%)	36 (9.6%)	0.10
- **C3**	25 (15%)	117 (31.4%)	**< 0.001**
- **Others**	8 (4.8%)	35 (12.3%)	**0.01**

III. Supplementary Table S2. Histopathological findings of DKD on renal biopsy – DKD versus NDKD plus DKD

Parameter	DKD Only (n=166)	NDKD plus DKD (n=110)	p-value
**Histological Class**			
- Class I	8 (4.8%)	2 (1.8%)	0.19
- Class IIA	20 (12%)	15 (13.6%)	0.69
- Class IIB	9 (5.4%)	9 (8.2%)	0.35
- Class III	64 (38.6%)	53 (48.2%)	0.11
- Class IV	65 (39.2%)	31 (28.1%)	0.38
**LM-Glomerular changes**			
- GBM Thickening	49 (29.5%)	54 (49%)	0.003
- Mild mesangial expansion	117 (70.4%)	45 (40.9%)	< 0.001
- Severe mesangial expansion	15 (9.3%)	25 (22.7%)	< 0.001
- KW lesions	55 (33.1%)	52 (47.2%)	0.03
- No DM related glomerular lesions on LM	8 (4.8%)	2 (1.8%)	0.19

LM: Light microscopy, GBM: Glomerular basement membrane;
KW lesions: Kimmelstiel-Wilson lesions
